# Electromagnetic Correlates of Musical Expertise in Processing of Tone Patterns

**DOI:** 10.1371/journal.pone.0030171

**Published:** 2012-01-18

**Authors:** Anja Kuchenbuch, Evangelos Paraskevopoulos, Sibylle C. Herholz, Christo Pantev

**Affiliations:** 1 Institute for Biomagnetism and Biosignalanalysis, University of Münster, Münster, Germany; 2 Montreal Neurological Institute, McGill University, Montreal, Quebec, Canada; University of Manchester, United Kingdom

## Abstract

Using magnetoencephalography (MEG), we investigated the influence of long term musical training on the processing of partly imagined tone patterns (*imagery condition*) compared to the same perceived patterns (*perceptual condition*). The magnetic counterpart of the mismatch negativity (MMNm) was recorded and compared between musicians and non-musicians in order to assess the effect of musical training on the detection of deviants to tone patterns. The results indicated a clear MMNm in the perceptual condition as well as in a simple pitch oddball (control) condition in both groups. However, there was no significant mismatch response in either group in the imagery condition despite above chance behavioral performance in the task of detecting deviant tones. The latency and the laterality of the MMNm in the perceptual condition differed significantly between groups, with an earlier MMNm in musicians, especially in the left hemisphere. In contrast the MMNm amplitudes did not differ significantly between groups. The behavioral results revealed a clear effect of long-term musical training in both experimental conditions. The obtained results represent new evidence that the processing of tone patterns is faster and more strongly lateralized in musically trained subjects, which is consistent with other findings in different paradigms of enhanced auditory neural system functioning due to long-term musical training.

## Introduction

Musical training has been recognized as an important model in cognitive neuroscience for experience-dependent plasticity and efficiency of processing in the auditory cortex [1]–[6]. Various cross-sectional studies showed differences between musicians and non-musicians, indicating pronounced effects of long-term musical training on cortical processing and plasticity [7]–[12] while training studies provide experimental evidence of the influence of short-term musical training [13]–[16]. Both approaches have advantages and limitations. Whereas group comparisons can indicate effects of long-term training but do not allow drawing direct conclusions about ‘nature’ or ‘nurture’, training studies allow causal inference but are limited regarding the length of the experimentally controlled training. However, converging evidence of both cross-sectional and training studies indicates that while pre-existing differences may make some contribution, training plays a very important role in the differences observed in musicians compared to musically untrained subjects, thus validating comparisons of musicians and non-musicians are a very good model for long-term training effects.

The mismatch negativity (MMN, sometimes also termed MMNm in MEG studies) is an event-related component that reflects the detection of violations of previously encoded regularities in auditory stimuli [17] and that has been used to investigate the neuronal underpinnings of auditory processing [17]–[19]. The MMN response is reliably elicited in oddball paradigms, in which the same sound (standard) is repeatedly presented. Infrequently occurring deviant sounds that differ from the standard in one or more features such as frequency, loudness or duration evoke the mismatch response. The main sources of the MMN are located in secondary auditory cortical structures [19]. Although the process reflected in the MMN is considered pre-attentive and is even reported during sleep and in coma patients [17], the MMN can nonetheless be modulated by attention [20], [21].

Recent studies indicate that the MMN(m) is not only evoked by sounds that deviate from the standard regarding simple physical features (pitch, loudness or duration, [22]), but also by deviant tones in short melodies or tone patterns [23], [24] and by deviants that violate complex rules in the auditory input [25]–[27]. Musical training has been shown to enhance the MMN(m) response and affect the encoding of complex auditory stimuli such as short melodies [7], [28] and tone patterns [9], [12], [29]–[32], whereas it does not seem to enhance the MMN(m) response and the encoding of regularities based on simple stimulus features [7], [28], [30], [33], [34].

Neuronal activity in auditory cortices can also be evoked in the absence of sensory (auditory) stimulation during auditory imagery [35]–[42]. Furthermore it has been shown that musical expertise enhances the ability for auditory imagery [8], [43]. In a behavioral study [43] musicians outperformed non-musicians in a musical imagery task as well as in a non-musical auditory imagery task.

A recent study by Herholz et al. [8] demonstrated MMNm responses in professional musicians based on imagined melodies. In this study subjects listened to the beginning of a familiar melody, continued the melody in their mind and then compared a presented test tone to the expected imagined tone at this point of the melody. If the perceived and imagined tone did not match, a significant MMNm response was recorded in musicians, whereas non-musicians did not generate an MMNm response despite above chance behavioral performance. This clearly demonstrated that MMNm-like responses can be evoked based on auditory imagery. This suggests that the MMN represents a more general mechanism of regularity violation detection.

In the present study, we continue investigating the influence of long-term musical training on the processing of partly imagined tone patterns (imagery condition) compared to same perceived patterns (perceptual condition) by analysing the evoked MMNm to pattern deviants. In the study of Herholz et al. no perceptual condition was included and therefore no direct comparison between imagery and perceptual MMN could be performed. To our knowledge there are no other studies that compared imagery and perceptual MMN directly. Therefore the goal of this study was determining to what extent both components resemble each other and in which aspects they differ by comparing them directly. A further goal was to investigate the influence of musical training on the processing of tone patterns. The following hypotheses were made: (i) more pronounced MMNm in musicians within the imagery and perceptual conditions and no differences between musicians and non-musicians in a classical frequency oddball control condition (ii) smaller MMNm in the imagery than in the perceptual condition for both musicians and non-musicians, (iii) behavioral results of detecting deviant tones are related to the electrophysiological indicators of the deviant detection.

## Materials and Methods

### Subjects

Thirty-two subjects participated in the experiment. Five subjects had to be excluded from the final data analysis due to insufficient recording quality, excessive head movements, or insufficient quality of the model fit of their recorded data (three musicians, two non-musicians). The remaining 27 subjects, 13 musicians (mean age: 27.15; *SD*: 8.77; 4 males) and 14 non-musicians (mean age: 25.21; *SD*: 2.91; 5 males) were included in the final data analysis. Musicians were students of the Music Conservatory in Münster, Germany or professionals or had received extensive musical training since childhood (minimum ten years) and were still actively playing an instrument. Two of them reported to have absolute pitch (self-report), although this was not explicitly measured. Non-musicians had not received any musical training apart from basic compulsory music classes in school. All subjects were right handed as assessed by Edinburgh Handedness Inventory [44], had normal hearing as assessed by audiometry and provided written consent prior to their participation in the study. The study protocol was approved by the ethics committee of the medical faculty of the University of Münster and the study was conducted according to the Declaration of Helsinki.

### Stimuli and Procedure

**Figure 1:**
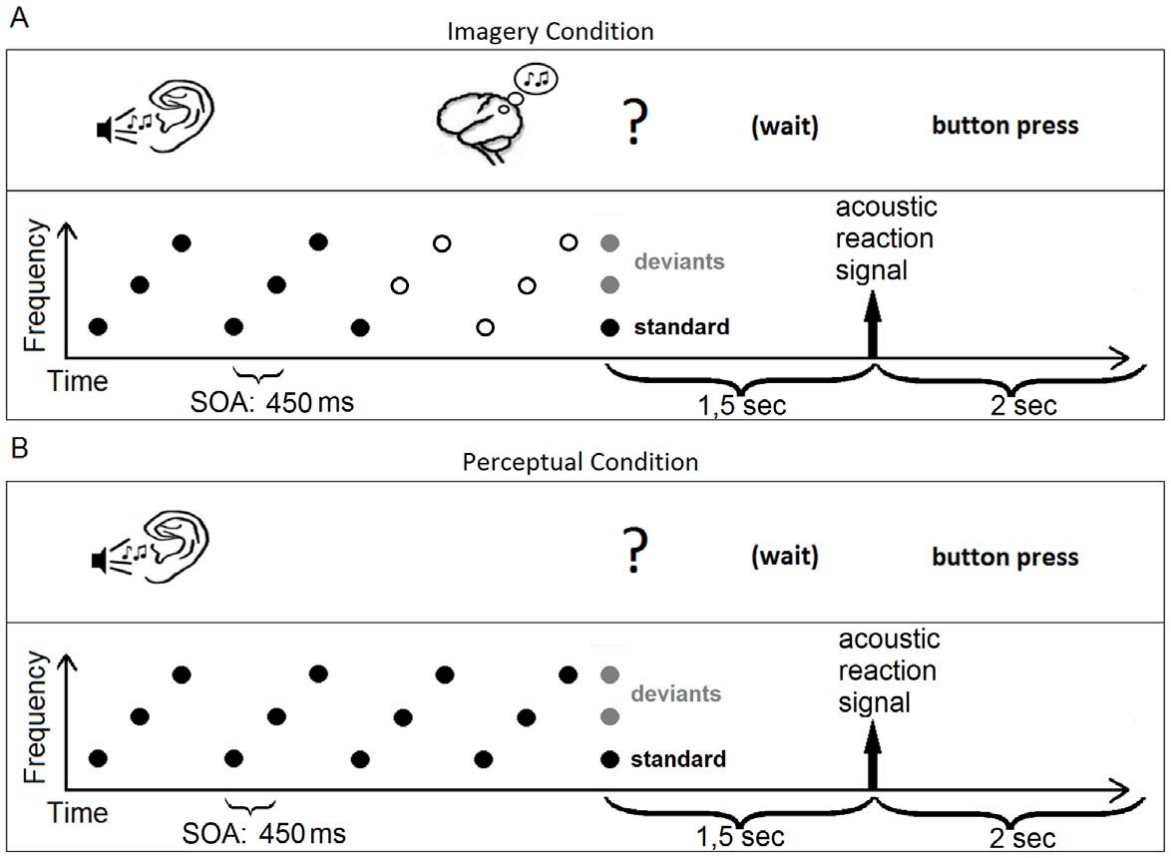
Graphical representation of a trial of the imagery condition (A) and the perceptual condition (B). In the imagery condition the tone pattern had to be continued in the imagination. The last tone in both conditions could either be the correct continuation of the melody *at that time point*, with a probability of 50% (standard, depicted by the black dot) or one of the other tones of the tone pattern, with a probability of 25%, respectively (deviants as depicted by the grey dots).

Three tone patterns were used in the experiment; each of them consisting of repetitions of an ascending tone pattern composed of three different sinusoidal tones. The tones were generated within one key (C-major) in 44100 Hz stereo and 32bit, and the notes of the three tone pattern corresponded to CEG, DFA and EGB in musical notation (range from lowest to highest tone 261.63 to 493.88 Hz). The duration of each tone was 300 ms including 10 ms rise and decay, and the interstimulus interval duration was 150 ms. In total, 192 trials were presented for each condition. In each trial of the perceptual condition one of the three possible tone patterns was repeatedly played. The length of the total tone sequence on individual trials varied between 10 and 13 tones. The tone sequence was followed by a 1.5 seconds break and a short sound prompting the subject to respond. The last tone of each sequence was the test tone. When prompted, subjects indicated via button press if they thought that the test tone represented a correct continuation of the pattern or not. A response was permitted within a two-second window following the prompt; early or late responses resulted in corrective visual feedback. The imagery condition differed from the perceptual condition in that the period between the seventh and last tones was replaced by silence. The length of this silent interval varied randomly between individual imagery trials, with lengths corresponding to the duration of two to five tones, resulting in four different lengths of the overall sequence that corresponded to the overall lengths of the sequences in the perceptual condition. An illustration of example imagery and perceptual trials is shown in figure 1. In both conditions the test tone was either the correct continuation of the pattern (standard tone) or one of the other two tones (deviant tone). Standard and deviant trials, respectively, occurred randomly with a probability of 50% (each of the two deviants with a probability of 25%) in both conditions.

In the control condition two tones were presented (1000 Hz and 1200 Hz) in a frequency oddball paradigm. The tones were presented as continuous stream with tone duration of 300 ms including 10 ms rise and decay and ISI of 150 ms. In total, 995 trials were presented. The probability of deviant occurrence was set at 0.2. At least 3 standards preceded each deviant. Subjects listened to the control condition while they were attending to a silent movie of their own choice. No behavioral measurements were recorded in the control condition.

The subjects participated in the study on two consecutive days. On the first day they completed 10 minutes of training of the perceptual condition followed by the recording of the perceptual condition in the MEG-Scanner. After a short break they took part in a 40 minute training session for the imagery condition. On the second day they did a short refresher training of the imagery condition of 10 minutes duration, followed by the recording of the imagery condition in the MEG Scanner. The same paradigms were used in the training phases and MEG recordings for both conditions, but no MEG data were recorded during training phases. During the training phases the subjects were provided with feedback if their answer was correct or not, whereas during MEG recordings they received no feedback. The control condition was recorded immediately after the imagery condition.

### MEG recordings

Magnetic fields were recorded with a 275 channel whole-head system (OMEGA, CTF Systems Inc, Port Coquitlam, Canada) in an acoustically and magnetically shielded room. MEG data were acquired continuously during presentation blocks with a sampling rate of 600 Hz. The subjects listened to the four (perceptual condition) or five blocks (four blocks imagery and one block control condition) with short breaks in between, during which they could relax. They were seated upright, and their head position was comfortably stabilized with pads inside the dewar. Stimuli were delivered via air conduction in plastic tubes at 60 dB SL above the individual hearing threshold, which was determined with an accuracy of 5 dB for each ear at the beginning of each MEG session for the different stimuli. The subject's alertness and compliance were verified by video monitoring. The subjects were instructed to minimize swallowing and blinking and to do so in between trials if possible.

### Behavioral measurements

Percentages of correct answers (hits and correct rejections) were averaged across the four blocks of the perceptual and of the imagery condition, respectively, for each subject. Scores were subjected to statistical tests for group analysis. Reaction time could not be taken into account because the subjects were prompted to react after a pause of 1.5 seconds in order to avoid muscle activity interfering with the MEG signal.

### MEG data analysis

The continuous data were separated in epochs of 600 ms, starting 100 ms before the last tone of each tone pattern in the perceptual or imagery condition (test tones) and every tone of the control condition and ending 500 ms after the tone onset. Epochs containing signal amplitudes >2.5 pT were considered artifacts and were excluded from averaging. Epochs were baseline corrected based on the 100 ms baseline before tone onset. Measurements of all four blocks of the perceptual and imagery condition, respectively, were combined in order to achieve the best signal-to-noise ratio possible. Standards and deviants were averaged separately and digitally filtered (high pass filter of 1 Hz and a low pass filter of 30 Hz). Averaged responses to standards were subtracted from averaged responses to deviants in order to acquire the difference response containing the MMNm in all three conditions.

In the analysis of the control condition two equivalent current dipoles (ECD) one in each hemisphere, were used to model the MMNm field, a technique justified by the dipolar distribution of the MMNm [45]. The ECDs were fitted simultaneously in a spherical volume conductor to each individual's peak of MMNm in the averaged difference response. Source waveforms for each of the subjects in each of the conditions were derived from the MEG data using the technique of signal space projection [46], thereby reducing the data to one source waveform for each hemisphere.

It was not possible to obtain a sufficient fit for the MMNm in all subjects in both the perceptual and imagery conditions, because in some cases, especially in the imagery condition, the difference between standards and deviant was not very pronounced. Therefore the more clearly pronounced N1 of the deviant waveform was used for source localization in all subjects in the perceptual and imagery condition. Although the auditory N1 and MMNm do not share the exact same source and same cognitive mechanisms [19] the spatial closeness of the sources of both components makes it possible to examine the MMNm component in the difference source waveform derived from the fit of the N1 in the averaged deviant response. This is a valid approach as the sources of the MMN and N1 in the auditory cortex are very close [19] and because the source space projection method is robust to slight displacements of sources [46]. It has also been adopted in other studies [7], [8], [28] where a reliable fit could not been obtained in all subjects. All dipoles included in the present analysis explained at least 85% of the magnetic field variance.

In order to ascertain that possible differences in the N1 amplitude were not mistaken for MMNm peaks, we compared the latencies of both components. A paired-sample t-test comparing N1 and MMNm latencies of the left (mean latencies of all subjects: MMNm peak: 164.5 ms, N1 peak: 124.0 ms) and right (MMNm peak: 165.6 ms, N1 peak: 125.6 ms) hemisphere in the perceptual condition reveal a highly significant difference between the latencies of those two components [left hemisphere: *t*(25) = 5.563, *p*<.001, right hemisphere: *t*(25) = 7.132, *p*<.001], showing that the MMNm peaks are not intermixed with the N1 peaks. There was no MMNm peak in the imagery condition, and therefore a corresponding comparison was not conducted for this condition.

Individual amplitudes and latencies of the MMNm were entered in statistical analyses. In all analyses the alpha level was 0.05, and tests were two-tailed. In order to estimate if the components differed significantly from zero, nonparametric bootstrapping (1000 resampling iterations) was applied to the group averaged waveforms for the MMNm in both hemispheres for all conditions [47]–[50]. The bootstrapping method estimates a confidence interval around the mean. Values outside of this confidence interval are considered significantly different from the mean. Accordingly, time windows in which the 95 percent confidence interval of the bootstrap around the averaged source waveform did not include zero values were considered to indicate significant deflections.

## Results

### Behavioral data

**Figure 2:**
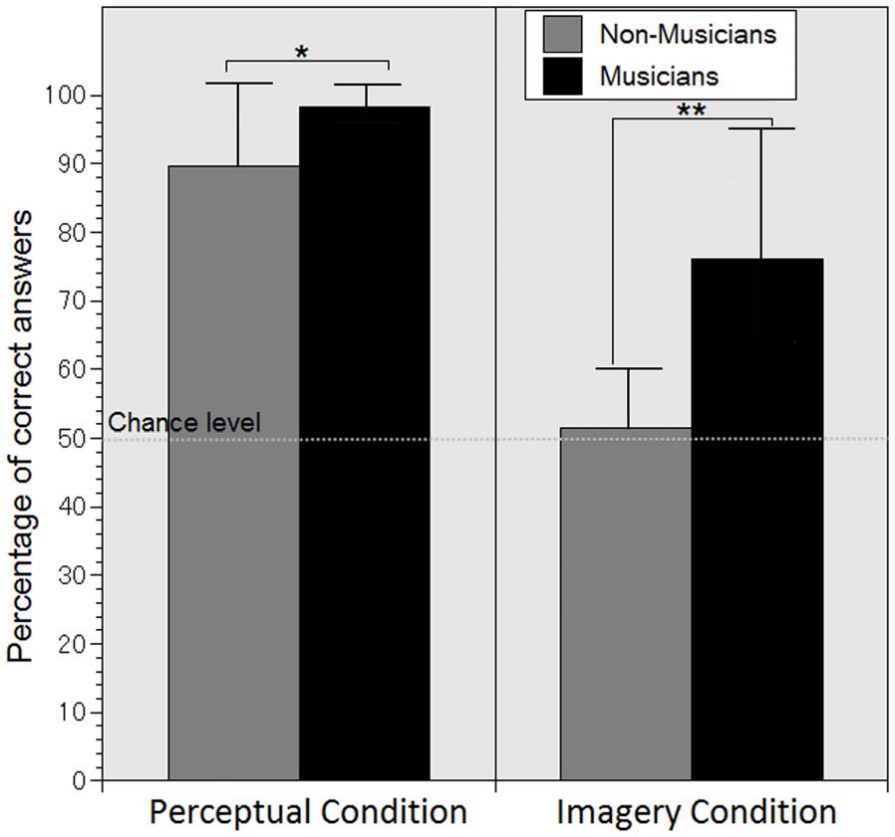
Mean correct answers in percent of the musicians and non-musicians for the imagery and the perceptual condition. The dashed line represents the chance level and the bar on the graphs represents the standard deviation. The between group difference in the perceptual condition is significant at p<.05 (indicated by *) and in the imagery condition it is significant at p<.01 (indicated by **).

As expected, musicians performed better in the behavioral task than the non-musicians in the perceptual condition [*t*(25) = 2.463, *p* = .026, independent sample *t* – test] as well as in the imagery condition [*t*(25) = 4.372, *p*<.001; independent sample *t* – test]. Musicians excelled in distinguishing between correct and incorrect tones in the perceptual condition, with an average score of 98±3% (SD) correct answers, and performed well in the more difficult task of the imagery condition, with an average score of 76±19% (SD) correct answers. In the perceptual condition, non-musicians performed above chance level but with slightly poorer score than musicians with 90±12% (SD) correct answers. In the imagery condition, non-musicians did not perform above chance level [*t*(13) = 0.681, *p*>.05; one – sample t–test], with only 51.5±8% (SD) correct answers. This indicates that the non-musicians were not able to distinguish between correct and incorrect tones in the imagery condition. The average of correct responses in both groups are shown in Figure 2.

### MEG data

**Figure 3:**
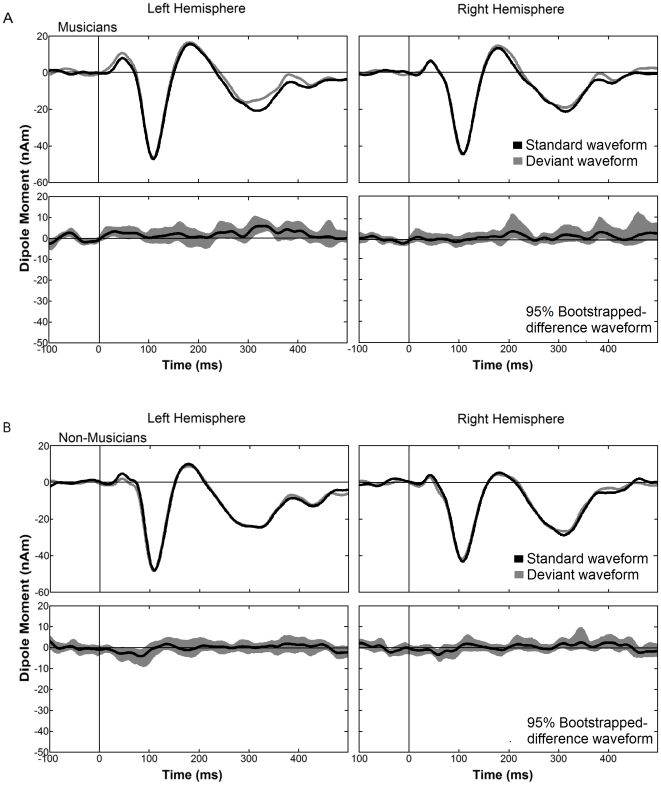
Grand averaged source waveforms of the imagery condition obtained from the individual dipole moment of MMN for musicians (A) and non-musicians (B). For each group the upper panels show the response to standard (black trace) and deviant stimuli (gray trace), and the lower panels show the difference waveforms (black trace) with 95% bootstrapped confidence intervals (gray shaded areas). Time windows in which the 95 percent confidence interval of the bootstrap around the averaged source waveform did not include zero values were considered to indicate significant deflections. In all panels the left hemisphere is presented on the left side and the right on the right.

Although musicians were behaviorally able to distinguish between correct and incorrect tones in the imagery condition we were unable to detect a corresponding effect in the electrophysiological data. In our data sets there was neither a difference between the responses to deviants and standards in the grand averaged source waveforms of the MMNm of either group as depicted in Figure 3, nor in the individual source waveforms of subjects in either group.

**Figure 4:**
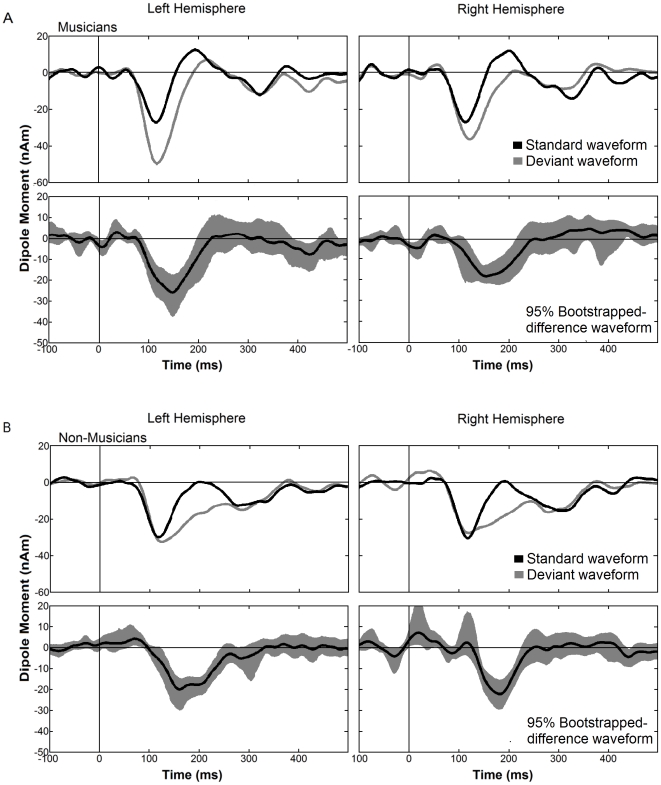
Grand averaged source waveforms of the perceptual condition obtained from the individual dipole moment of MMN for musicians (A) and non-musicians (B). For each group the upper panels show the response to standard (black trace) and deviant stimuli (gray trace), and the lower panels show the difference waveforms (black trace) with 95% bootstrapped confidence intervals (gray shaded areas). Time windows in which the 95 percent confidence interval of the bootstrap around the averaged source waveform did not include zero values were considered to indicate significant deflections. In all panels the left hemisphere is presented on the left side and the right on the right.

In contrast, clear MMNm responses were elicited in the perceptual condition. The grand averaged source waveforms, for the perceptual condition are shown in Figure 4. Both groups demonstrate significant deflections of MMNm to deviants as revealed by a nonparametric bootstrap analysis of the difference waveforms.

**Figure 5:**
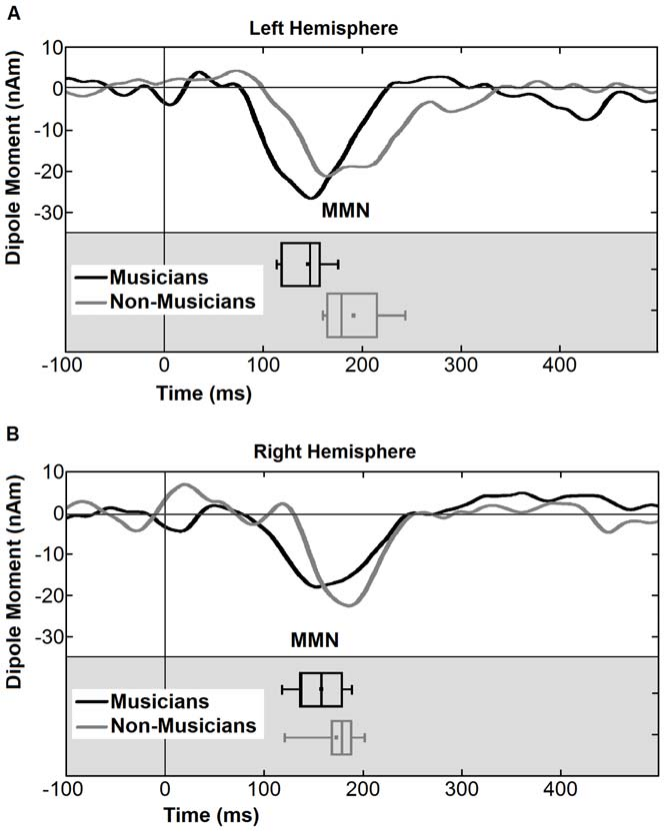
Grand averaged difference source waveforms, obtained from the individual dipole moment of MMN for musicians (black) and non-musicians (gray) for left (A) and right (B) hemispheres for the perceptual condition. Boxplots for each group and hemisphere are also presented and rotated so that they match to the latency as indicated on the x axis.

Amplitude and latency of the individual MMNm peaks in the perceptual condition were entered into two mixed model 2×2 ANOVA with group (musician and non-musician) as the between-subject factor and hemisphere (left and right) as the within-subject factor. For latency, we found a highly significant main effect of group [*F*(1,25) = 10.118, *p* = .004] and a significant interaction of group×hemisphere [*F*(1,25) = 4.886, *p* = .036] indicating that the MMNm response was earlier in musicians, especially in the left hemisphere (c.f. Figure 5). Analysis of the amplitudes did not reveal any significant main effects [Group: *F*(1,25) = 0.307 *p*< = .584; Hemisphere: *F*(1,25) = 0.785, *p* = .384] or interactions [Group×Hemisphere: *F*(1,25) = 0.866, *p* = .361], indicating that the two groups did not differ systematically in the amplitude of their MMNm as displayed in Figure 4 and 5. In order to examine the effect of different lengths of the sequences, we analyzed the averaged source waveforms of each of the four sequence lengths separately. However, there was no systematic effect of sequence length on the difference between the responses to deviants and standards.

**Figure 6:**
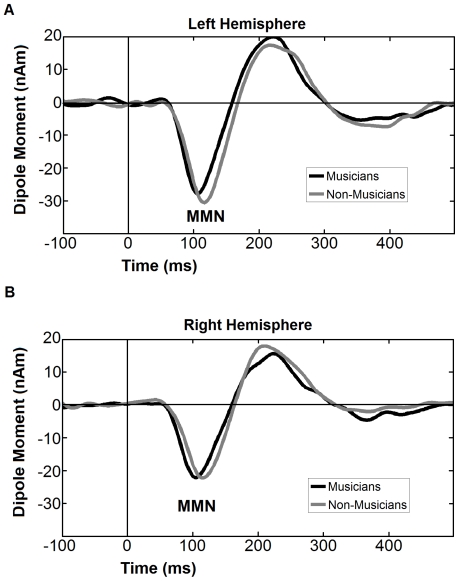
Grand averaged difference source waveforms, obtained from the individual dipole moment of MMN for musicians (black) and non-musicians (gray) for left (A) and right (B) hemispheres for the control condition.

As expected, in the control condition there was no significant difference between groups for the MMNm neither in peak amplitude nor in MMNm latency, as revealed by a mixed model 2×2 ANOVA with group (musician and non-musician) as the between-subject factor and hemisphere (left and right) as the within-subject factor [Group, latency : *F*(1,25) = 1.035 *p*< = .319; group amplitude: *F*(1,25) = 0.074 *p*< = .787 ] However, we found a main effect of hemisphere in amplitude [*F*(1,25) = 6.904 *p*< = .014], indicating that the MMNm in the left hemisphere is more pronounced than the right hemisphere. The grand averaged source waveforms are displayed in Figure 6.

## Discussion

In the present study, we investigated the influence of long-term musical training on auditory processing and mental imagery of melodic patterns by means of magnetoencephalography and of a corresponding behavioral task. We anticipated that detection of deviant tones would correspond to the electrophysiological indicators of deviance detection, such as in an MMNm response [18]. Despite above chance behavioral performance in the musicians' group in the imagery condition we were unable to replicate the imagery MMNm response demonstrated in a previous study of Herholz et al. [8]. However, we observed enhanced behavioral performance in the imagery task in musicians compared to non-musicians, thereby replicating the beneficial effect of long-term musical training on auditory imagery at the behavioral level of Herholz et al. [8]. In contrast to the imagery condition, we did find an MMNm response to deviant tones in the perceptual condition , which is in line with previous studies on encoding of higher-order regularities at the level of auditory cortex [51]–[56]. Whereas previous studies showed an effect of long-term musical training on deviant detection in complex tonal patterns [9], [29], [31], [32], [57] we did not find group differences for MMNm amplitude. The reason for the lack of differences is likely due to the task demands in the perceptual condition: The deviation to be identified in this condition was very obvious and easy to detect. Taken together, both the high amplitudes and the good performance are due to the low difficulty or obvious deviance in the perceptual condition, and the resulting ceiling effect likely makes it difficult to detect group effects in the MEG data. As expected, in the control condition we did not find group differences for MMNm amplitude or latency, however, we observed a more pronounced MMNm in the left hemisphere. This is an interesting finding and should be investigated in further experiments, but we will not expand on this because it was not the focus of our research. Nevertheless, as another main finding, we observed shorter MMNm latencies in musicians in the perceptual condition, especially in the left hemisphere. This is consistent with previous research that showed the superiority of musicians in processing complex auditory material [12], [30]–[32]. We discuss each of these findings in more detail in the following sections.

### Differences in neural activation in auditory imagery regarding familiarity

Neural activity during auditory imagery shares similar activation patterns with actual audition [35], [37]–[42], with differences in activation depending on the degree of familiarity of the imagined material. Familiar auditory material compared to unfamiliar auditory material in general elicits enhanced auditory processing [37], [58]. Most importantly, the imagination of familiar auditory material is related to enhanced activation of the auditory association cortex (Brodmann's area 22) and frontal cortices compared to unfamiliar auditory material [35], [37].

In the present study the tone patterns used were first introduced to the subjects during the experiment. This is unlike the study of Herholz et al. [8], in which familiar melodies were used that were meaningful to the subjects, that were well-known since childhood and that were represented in long-term memory. Therefore, the newly introduced tone patterns of our study were less salient for the subjects than the familiar melodies used in Herholz et al [8]. Because familiarity impacts the amplitude of activation in secondary auditory cortex during mental imagery, secondary auditory cortices might not have been engaged to the same extent during the imagery of the new tone patterns as during imagery of the familiar tunes in the previous study. Thus, although the neuronal representation of the patterns during imagery were sufficient for above chance behavioral performance (in musicians), it still might have been an insufficient basis for the (pre-attentive) mismatch negativity. Also, whereas all deviants in the study of Herholz et al. [8] were in key, they were not necessarily part of the familiar melodies. In contrast, in the present study both deviant and standard tones were part of the pattern. Therefore standards and deviants in the present study were easier to confuse, and deviants were less distinct and salient regarding their pitch than in the previous study, which might have led to a decreased response on the neuronal level.

The clear and statistically significant effect of group on the behavioral performance in the imagery task is consistent with previous results showing enhanced auditory imagery capabilities in musicians compared to non-musicians [8], [43]. Whereas previous studies used familiar material (familiar songs and everyday sounds), the present study is the first to show that the effects of long-term musical training on auditory imagery also transfer to unfamiliar and rather abstract tonal material, suggesting a general enhancement of auditory imagery capabilities in musicians. However, this difference between musicians and non-musicians may also be interpreted as the ability of musicians to acquaint themselves with new tonal material more quickly than non-musicians due to their long term musical training even though they were as unfamiliar with the auditory material as the non-musicians. Because the stimuli were introduced during the training session, subjects encountered the stimuli before the MEG recordings, allowing for a brief learning period. However, it could also be argued that our stimuli were arpeggiated chords and musicians should be more accustomed with arpeggiated chords in general, although not necessarily with chords composed of sinusoidal tones. We will now discuss the issue of timing in imagery, which is another plausible reason for better performance in musicians.

### Keeping in time is essential for musicians

In order to successfully detect deviant tones, it is necessary to maintain a constant tempo such that the comparison is made between the *correct imagined tone at the correct time* with the tone that was presented. Otherwise, if the tempo during imagery was incorrect, the deviant might be perceived as a correct continuation of the tone pattern, or the standard might be perceived as an incorrect continuation, thereby resulting in diminished performance. For example, whereas a C would be a deviant at the end of the pattern “CEGCEGCE*C* ” it would be a standard in a pattern that contains one more tone before the test tone (“CEGCEGCEG*C* ”). Therefore, if subjects increased or decreased the tempo during the imagery interval, correct deviant detection would be impossible. Keeping time in a musical meter is an essential part of musical education, which would not be provided to the non-musicians. Therefore, we can assume that the musicians' better behavioral performance is partly due to their better ability to keep the time in their mind. This is consistent with findings indicating that rhythm and meter are much more salient for musicians, because rhythm and meter go along with expectancy and structuring of the music. Vuust et al. [59] compared professional musicians (jazz players) and non-musicians and showed that responses to violations of a certain meter were more pronounced in musicians than in non-musicians. Likewise, in a study by van Zuijen et al. [32] musicians showed better encoding of numerical regularities without attention, reflecting the importance of beat tracking in music.

### Laterality of auditory processing in musicians

An important and interesting finding of this study was the effect of musical training on the pattern MMNm latency: In the perceptual condition there was no difference in MMNm amplitude between musicians and non-musicians, as was the case in the control condition. The latter was included in order to reveal the absence of influence of long term musical training on the general ability of human auditory cortex to detect salient frequency violations in auditory stimuli. However, there was a significant difference between groups in the latency of the MMNm in response to pattern violations, which occurred earlier in musicians than in non-musicians, especially in the left hemisphere. We will discuss this finding in the following with respect to both the laterality and the interpretation of latency differences of MMNm responses.

Generally speaking, language processing in humans is more strongly left-lateralized while music processing is more strongly right-lateralized [18], [60]. However, there is evidence that some aspects of music and tone processing are left-lateralized as well, depending on sound parameters such as the meter, sound familiarity, top down modulation and musical expertise [9], [59], [60]. In a training study with Morse codes, A. Kujala et al. [61] showed that the lateralization of processing shifted from the right to the left hemisphere as the Morse code tone pattern became meaningful to the learners through training [61]. In our study, we found an effect of musical expertise on the latency of the MMNm in the perceptual condition, where the group difference was especially pronounced in the left hemisphere. A similar effect, albeit on MMNm amplitude, was found by Vuust et al. [59] for MMNm responses to musical meter violations in musicians. Furthermore, in two recent studies by Herholz et al. [9], [29] musicians showed enhanced and more left-lateralized MMNm responses to violations of tone patterns that were embedded in an oddball paradigm. In these studies, regular and deviant patterns differed regarding the number of tones in the pattern, a salient temporal regularity. Although we did not test for perception of meter behaviorally, the triplet-structure of the present stimulation also had a very salient temporal structure and might even have induced the perception of a Waltzing meter. In a recent study, Boh et al. [57] observed a left-lateralization of the MMNm only in the tone pattern that could be structured according to a triplet meter. Accordingly, one possible interpretation for our results might also be an additional recruitment of left-lateralized networks involved in processing of metric information or temporal structures in musicians. This possible interpretation should be investigated in future studies that are specifically designed for testing it directly. Taken together, the results of the current study provide further indication that long-term musical training affects the lateralization of processing of pitches towards faster processing in the left hemisphere when there is a strong rhythmic or temporal component to the tonal stimulation.

### MMNm latency

The main group difference in the present study regarding processing of tone patterns was not regarding the amplitude of the MMNm, but instead regarding the latency of the MMNm response, with an earlier MMNm in the musicians, especially in the left hemisphere. This indicates that musicians were able to process the acoustic information more efficiently and faster than non-musicians, which is most probably due to their musical training. Previous research suggests that the dependency of amplitude, latency and violation magnitude is the following: With increasing magnitude of stimulus change, the MMNm peak latency decreases and the amplitude increases, and earlier latency signifies increased processing speed [17], [18]. There are other factors which modulate MMNm amplitude and latency such as attention, expectation, memory span and features of the stimuli, e.g. stimulus length and stimulus onset asynchrony [17]–[19]. However, little research has examined the influence of long term musical training on MMN(m) latency, although some research shows latency differences along with amplitude differences [7], [62], [63]. For example, Nikjeh et al. [62] investigated the influence of long-term musical training on the processing of pure (sinusoidal) tones, harmonic rich tones and speech syllables. In musicians, the latencies of MMN responses to harmonic and pure tones are significantly shorter than in non-musicians, while the MMN latencies for speech syllables did not differ significantly between groups. Similarly, the results of a study by Fujioka et al. [7] using polyphonic melodies as stimuli also show earlier and more left-lateralized MMNm responses in musicians, similar to the results of the present study. However, due to methodological reasons, Fujioka et al. [7] did not statistically confirm this finding. Also, in a study by Brattico et al. [58] shorter MMN latencies to pitch deviants in ascending tone patterns were found for musicians compared to non-musicians, whereas the groups did not differ regarding MMN amplitudes.

The reason for lack of group effect on MMNm amplitude in the present study could be explained by a ceiling effect, as the stimulus change (in the perceptual condition) was obvious and was identified by both groups in the behavioral measure. Still, we found a difference between musicians and non-musicians in the behavioral performance and this is reflected in a group difference for MMNm latency. Thus, in cases where MMNm amplitude does not distinguish between groups, latency can be a more sensitive indicator of processing advantages due to long-term musical training.

### Conclusion

This study provides evidence for the influence of musical training on behavioral correlates of auditory imagery of tone patterns, and on both behavioral and neuronal correlates of auditory processing of perceived melodic patterns. Musicians show faster and more left-lateralized processing of deviants in short tone patterns, suggesting faster neuronal processing of relevant auditory information. The results demonstrate that MMNm latency is a more sensitive marker for differences in early auditory processing between musicians and non-musicians than amplitude, and that it is therefore worthwhile to measure latency effects in future investigations of plasticity effects, for example of long-term musical training.
